# An Experiment-Based Profile Function for the Calculation of Damage Distribution in Bulk Silicon Induced by a Helium Focused Ion Beam Process

**DOI:** 10.3390/s20082306

**Published:** 2020-04-17

**Authors:** Qianhuang Chen, Tianyang Shao, Yan Xing

**Affiliations:** Jiangsu Key Laboratory for Design and Manufacture of Micro-Nano Biomedical Instruments, School of Mechanical Engineering, Southeast University, Nanjing 211189, China

**Keywords:** focused helium ion beam, ion dose, beam energy, silicon substrate, damage profile

## Abstract

The helium focused ion beam (He-FIB) is widely used in the field of nanostructure fabrication due to its high resolution. Complicated forms of processing damage induced by He-FIB can be observed in substrates, and these damages have a severe impact on nanostructure processing. This study experimentally investigated the influence of the beam energy and ion dose of He-FIB on processing damage. Based on the experimental results, a prediction function for the amorphous damage profile of the single-crystalline silicon substrate caused by incident He-FIB was proposed, and a method for calculating the amorphous damage profile by inputting ion dose and beam energy was established. Based on one set of the amorphous damage profiles, the function coefficients were determined using a genetic algorithm. Experiments on single-crystalline silicon scanned by He-FIB under different process parameters were carried out to validate the model. The proposed experiment-based model can accurately predict the amorphous damage profile induced by He-FIB under a wide range of different ion doses and beam energies.

## 1. Introduction

The helium focused ion beam (He-FIB) is widely used in the field of micro-nano structure fabrication because of its excellent processing performance. Compared with the traditional focused gallium ion beam, the He-FIB system using a gas field ion source (GFIS) has a higher resolution and brightness [1]. At present, He-FIB can be used in many aspects through an advanced process, such as TEM sample preparation, mask repair [2], nanostructure deposition [3,4], nanolithography, and nanostructure milling.

He-FIB is widely used to fabricate micro-nano structures on two-dimensional (2D) materials, such as graphene [5,6] and silicon membranes. Zheng successfully applied He-FIB to the post-fabrication modification of nanomechanical resonators and milled a linear of holes along the length of string [7]. Nanoelectronic devices with feature separations of sub-10 nm can be deposited by selectively scanning the He-FIB on graphene in a pattern defined by bitmap [8]. He-FIB is also used in resist-based lithography. Winston has fabricated sub-10 nm nanostructures on resists such as hydrogen silsesquioxane on silicon by resist-based helium-ion-beam lithography [9,10]. Since amorphous damage occurs in these nanoscale structures and devices during fabrication, it is necessary to control side effects.

The sputter yield is an important indicator in milling process. In 2D materials, the total sputter yield includes forward sputtered yield and back sputtered yield as part of the injected ions penetrates the material. However, in bulk materials, only the surface atoms are sputtered, since most of the injected ions are implanted into the substrate [11]. However, during the milling process, the injected helium ions cause additional damage in the substrate. In crystalline materials, including silicon and diamond, the implanted helium ions mainly lose energy via electronic interactions and the remainder through nuclear collision events. The target atoms displace from their origin lattice during the collisions, which causes displacements and voids. Besides, bubbles are formed with the implantation of helium ions. With the defects accumulating, the material completes the amorphization transformation [12]. The amorphous area extends with the increasing ion dose [13]. In bulk silicon, the implantation of helium ions also leads to surface swelling [14]. The amorphization, bubbles and swelling result in a significant reduction in the material density [15]. These changes of physical properties and inner structure have a great impact on the use of He-FIB for the manufacture of sensors on bulk silicon material. Therefore, it is necessary to establish a prediction model of the amorphous damage distribution. Besides, according to the cases of auxiliary gas or laser-assisted milling, although the sputtering yield shows an obvious increase, the damage profiles are similar to the profile of no auxiliary milling [16,17]. Hence, the prediction model can be a general solution for the above situation. The amorphous damage is controlled by the process parameters of He-FIB, including ion dose, beam energy, and beam current [18]. For example, changes in ion dose affect the overall range of the amorphous damage. Livengood compared the amorphous damage characteristics that occurred in a single-crystalline silicon film and bulk material due to incident high energy He-FIB [11]. The experiment showed that the pore diameter of the lower surface of the film material was larger than the pore diameter of its upper surface. Moreover, the damage range inside the bulk material was larger than the damage range visible on the bulk material surface. The ion current had almost no effect on the amorphous damage induced by He-FIB in the range where the ion current varied from 0.7 to 4.3 pA [18]. Nevertheless, the current research on He-FIB processing includes only qualitative analysis and simple quantitative analysis, and there is still a lack of quantitative analysis of the changes of amorphous area with process parameters.

**Figure 1:**
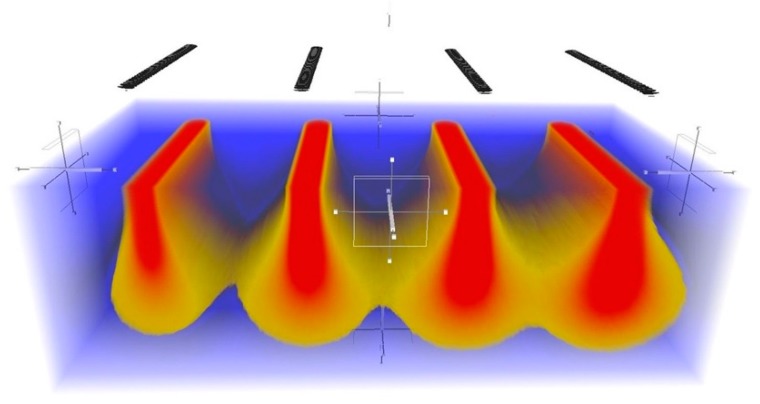
Damage of the silicon substrate caused by incident He-FIB in Monte Carlo simulation.

There are two main simulation methods for He-FIB processing: molecular dynamics and the Monte Carlo method. Molecular dynamics analysis is mainly used to study the mechanism of damage formation. It can be used to simulate the early nucleation of helium atoms in solid material [19,20,21], and it can also be used to simulate the effect of temperature on the processing damage induced by He-FIB [22]. However, in molecular dynamics simulations, the size of the substrate is limited to a range of tens of atoms, so damage formations with a size of several hundred nanometers cannot be simulated. In contrast, the Monte Carlo method uses the idea of probability to solve computational problems of simulating the incidence of numerous helium ions [23]. SRIM [24] is a general tool for ion irradiation based the Monte Carlo simulation, and it is widely used to obtain the energy distribution and the sputtering yield of FIB processing. However, it lacks a clear relationship between defects and amorphous formation. Besides, although it can customize the beginning position of injected ions with an input file, the procedure is complicated and the number of input ions cannot exceed 100 thousand, which makes it difficult to simulate line scanning directly [24,25]. KSOME [26] has realized the simulation of the formation and migration of bubbles and holes in the substrate, but it cannot simulate amorphous damage in detail. Figure 1 illustrates the general damage information caused by incident helium ions by line scan. The black lines denote the scanning path with different ion doses. The blue part is the crystalline area, the red part is the amorphous area and the yellow part is the defects area. As the Monte Carlo model of focused helium ion beam processing still has several limitations, it is helpful to obtain the amorphous damage induced by He-FIB by a purely empirical prediction model.

This study designed and carried out a series of experiments of He-FIB incident on a single-crystalline silicon substrate. The changes of amorphous damage with the ion dose and beam energy were clarified. Considering particle-solid interactions during the He-FIB process, an experiment-based damage profile function (DPF) was proposed. The genetic algorithm was used to solve the DPF coefficients corresponding to the amorphous damage profile at line doses of 0.01, 0.02, 0.03, 0.04 and 0.05 nC/μm and the beam energy between 10 keV and 35 keV. As a function of process parameters, the DPF can predict the amorphous damage profile by inputting ion dose, beam energy, and beam spot. Finally, experimental results were compared with the results calculated by DPF. This function can accurately describe the extension of amorphous damage with ion doses above 0.01 nC/μm and is suitable for the prediction of the amorphous damage profile of a single-crystalline silicon substrate when mainstream He-FIB equipment is used to apply a single-pixel spot or line scan to the substrate.

## 2. Experiments

In this work, a CZ-grown silicon wafer with <100> orientation was selected. Before conducting experiments, the samples were ultrasonically cleaned in acetone at room temperature for 15 min, rinsed with de-ionized water, and finally dried at 120 °C for 30 min. A Zeiss Orion NanoFab was selected to generate and implant helium ions into the silicon substrate along single-pixel lines.

**Figure 2:**
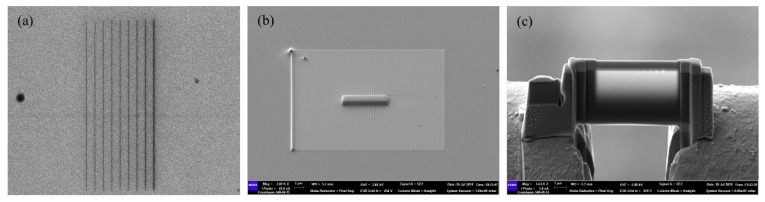
The process of fabrication and preparation of cross-sections of bulk silicon scanned by He-FIB with line doses of 0.003, 0.0045, 0.006, 0.0075, 0.01, 0.02, 0.03, 0.04 and 0.05 nC/μm at beam energy of 25 keV: (**a**) Helium ion microscope image of bulk silicon scanned by He-FIB; (**b**) Image of bulk silicon after platinum layer deposited by FIB; (**c**) The lamella welded on the copper grid.

It is known that the beam current has little influence on the damage profile when it is at a low level, whereas the beam energy has an obvious impact on the damage area. In this study, the experiments were performed with a set of beam energies, including 10, 15, 25 and 35 keV. For each energy level, five ion doses in the range of 0.01 to 0.05 nC/μm were selected to observe the formation and diffusion of defects and amorphous topography in silicon bulk. Before each experiment, the beam current was adjusted to 1.6 pA by adjusting helium pressure. Figure 2a shows the lines fabricated by helium ion beam of 25 keV beam energy.

A Zeiss Crossbeam 540 was used to prepare the transmission electron microscope (TEM) sample. To avoid the implantation of other ions, a 200 nm platinum layer was deposited first on the silicon surface by electron beam, and then the layer was deposited to 1 μm by gallium ion beam. Figure 2b shows the platinum layer deposited by FIB. After milling and pre-thinning of the backside and frontside, the lamella was extracted and shifted to the copper grid, as Figure 2c shows. Finally, the cross section of the damage profile was observed by FEI Talos F200X.

**Figure 3:**
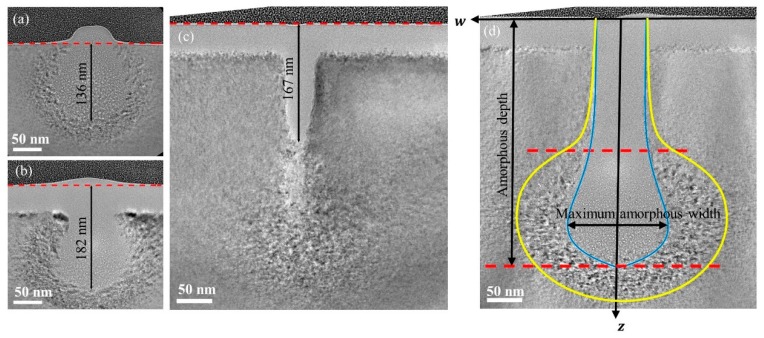
TEM images of cross-section samples of silicon substrate treated with He-FIB: (**a**) TEM image with an ion dose of 0.03 nC/μm at a beam energy of 10 keV; (**b**) TEM image with an ion dose of 0.03 nC/μm at a beam energy of 15 keV; (**c**) TEM image with an ion dose of 0.0075 nC/μm at a beam energy of 35 keV; (**d**) TEM image with an ion dose of 0.035 nC/μm at a beam energy of 35 keV.

Figure 3 shows cross-section TEM images of the silicon substrate with a dose of 0.03 nC/μm at beam energies of 10 and 15 keV and cross-section TEM images with 0.0075 and 0.035 nC/μm at beam energy of 35 keV. The amorphized part and dislocation part in the silicon are bright gray and dark gray, respectively. There forms a clear boundary between these two parts. This boundary line is the amorphous damage profile as marked by a blue line in Figure 3d. The region between the blue line and yellow line is the dislocation area. In this area, a different concentration of defects occurs according to the number of nuclear collisions [14,27]. As shown in Figure 3d, the coordinate system is defined by taking the center of the ion beam as the origin, the direction of incidence as the *z*-axis, and the surface of the substrate as the *w*-axis. The straight line where the *w*-axis is located is the sample surface, which is denoted by a red dotted line in Figure 3a–c. The characteristic dimensions of amorphous depth and maximum amorphous width are displayed in Figure 3d. In Figure 3b–d, there appears an amorphous silicon layer with a thickness of about 40 to 50 nm on the sample subsurface because of the long exposure by the helium ion beam imaging during the milling process.

Figure 3a,b show the slight change of amorphous damage profile when the beam energy increases from 10 keV to 15 keV with a 1.6 pA beam current and an ion dose of 0.03 nC/μm. The depth of the amorphous area increases by about 34% from 136 nm to 182 nm, but its lateral expansion is not obvious. It can be seen that the beam energy may only affect the depth of the amorphous damage. Figure 3c,d report the change of amorphous damage profile with increasing dose from 0.0075 nC/μm to 0.035 nC/μm at a fixed beam energy of 35 keV. When the ion energy is 0.0075 nC/μm, the amorphous damage profile appears ditch shape with a high aspect ratio. The depth and width of amorphous damage increase with the injection of helium ions. The amorphous area begins to appear obvious segmented features when the amorphous area continues to expand as shown in Figure 3d. The upper region of the amorphous damage still maintains a high aspect ratio, while the lower part greatly expands in the width direction. The upper and lower regions are separated by a red dotted line as shown in Figure 3d, which are denoted as the high-energy damage region and low-energy damage region, respectively, in this work. Similarly, the amorphous sections of other doses and beam energies can also be divided into these two regions.

The ions lose their energy in the substrate due to inelastic impact with electrons and elastic impact with atom nuclear. The defects caused by the elastic impact process contributes to the amorphization. Therefore, the amorphous region depends on the trajectories of helium ion beam and the interaction volume of nuclear energy loss. According to Biersack’s center of mass collision model, the scattering angle of injected helium ions is determined by the following three factors: the atomic number of the target atom, the energy of helium ion, and the impact parameter [24]. In this paper, the target atom is silicon. Hence, only the energy of helium ions affects the scattering angle under the same impact parameter. Besides, the scattering angle decreases monotonically with increasing energy, and large angle scattering rarely occurs on high energy helium ions. Therefore, the amorphous profile of the high-energy damage region has a high aspect ratio and slow lateral extension due to the high energy and similar incident angles of incident helium ions. In contrast, the low energy of ions in the low-energy damage region increases the proportion of large angle scattering, and eventually a droplet like damage region is formed. Based on the experimental results and theoretical assumptions, an empirical equation can be established to predict the damage profile of other beam energies and ion doses.

## 3. Methods

### 3.1. Damage Profile Function

**Figure 4:**
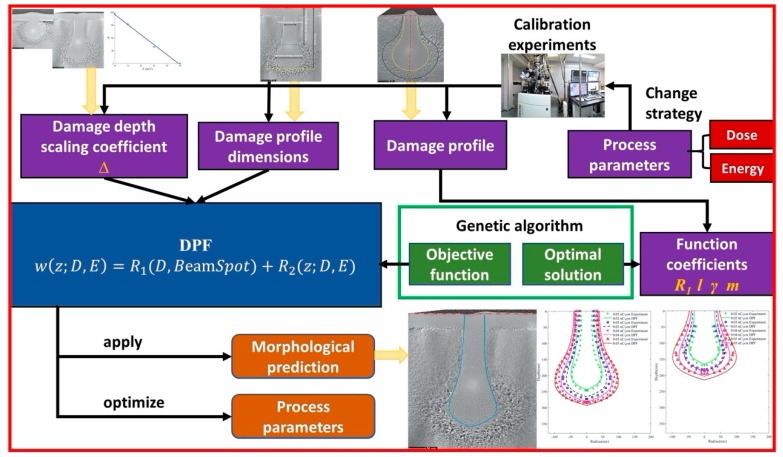
Establishment of DPF and its application process for amorphous damage profile prediction.

Based on the changes of the amorphous damage profile of single-crystalline silicon under incident He-FIB with process parameters, an empirical function describing the amorphous damage profile was established. Figure 4 shows the establishment of DPF and its application process for amorphous damage profile prediction. In addition to the ion dose and beam energy, the ion beam distribution also affects the amorphous damage profile. The beam spot directly affects the amorphous damage width on the substrate surface. The He-FIB injects into the substrate at normal incidence in a line scan mode, so the amorphous damage on the cross-section is symmetrical about the *z*-axis. This function only simulates the profile on one side of the *z*-axis. As a function of the half-width *w* of amorphous damage with respect to depth *z*, the DPF is as follows:

(1) $$w\left(z;D,E,BeamSpot\right)=R_{1}\left(D,BeamSpot\right)+R_{2}\left(z;D,E\right),$$

where *D* is the ion dose and *E* is the beam energy. The depth value when the amorphous damage half-width *w* is reduced to zero is defined as *z_max_*. The domain of the definition of DPF ranges from zero to *z_max_*. The DPF consists of two parts: high-energy damage calculation and low-energy damage calculation.

The high-energy damage calculation mainly reflects the influence of beam spot and ion dose on the high-energy damage region, as shown in Equation (2):

(2) $$R_{1}=\varphi \left(BeamSpot\right)\cdot s\left(D\right),$$

where *φ* is the beam spot influence coefficient function, and *s* is the dose influence coefficient function. The high-energy damage calculation only includes the calculation of surface damage width. The beam spot influence coefficient function represents the effect of beam spot on surface damage width. As shown in Equation (3), we consider that the effect of beam spot on the surface damage width is linear, and the values of *α*_1_ and *α*_2_ in this experiment are shown in Table 1. The functional relationship between the ion dose and the surface damage width can be considered to be a logarithmic relationship, as shown in Equation (4).

(3) $$\varphi =\alpha_{1}\times BeamSpot+\alpha_{2},$$

(4) $$s=\alpha_{3}\times \ln D+\alpha_{4}.$$

The low-energy damage calculation describes the effect of beam energy and ion dose on the low-energy damage region:

(5) $$R_{2}=\gamma \left(D\right)\cdot \left(1-\frac{z+\Delta \left(E\right)}{l\left(D\right)}\right){\left(\frac{z+\Delta \left(E\right)}{l\left(D\right)}\right)}^{m\left(D\right)}$$

where *l* is the damage depth coefficient function; *γ* is the maximum damage width coefficient function; *m* is the depth coefficient function of the maximum damage width; and Δ is the damage depth scaling coefficient function. The low-energy damage calculation describes the half-width change trend and depth of the amorphous damage profile. The relationship between the coefficients *l*, *γ* and *m* with respect to the ion dose *D* is shown in Equations (6)–(8), and the relationship between the coefficient ∆ and the beam energy *E* is shown in Equation (9):

(6) $$l=\beta_{1}\times \ln D+\beta_{2}$$

(7) $$\gamma =\beta_{3}\times D+\beta_{4},$$

(8) $$m=\beta_{5}\times D+\beta_{6},$$

(9) $$\Delta =\beta_{7}\times E+\beta_{8}.$$

From the increase in damage depth with ion dose, when the ion dose is less than 0.01 nC/µm, the damage depth increases rapidly with the ion dose. However, when the ion dose exceeds 0.01 nC/µm, the increase in the damage depth gradually slows down and finally stabilizes. Therefore, a logarithmic function form is used to fit the increase in the damage depth coefficient *l* with the ion dose *D*. The maximum damage diameter coefficient *γ* and its depth coefficient *m* have a linear relationship with the ion dose *D*.

The damage depth scaling coefficient Δ determines the overall shape of the profile. As shown in Figure 3a,b or Figure 3d, the low-energy damage regions of amorphous damage at different beam energies appear to have similar damage profiles. Therefore, the amorphous damage profile of the low-energy ion beam incident on the substrate can be regarded as a part of the amorphous damage profile caused by the high-energy ion beam. From the damage profiles of several beam energies, the amorphous damage depth is approximately proportional to the beam energy. Because the stable acceleration voltage of the experiment equipment used in this study is 10–35 kV, the amorphous damage profile at the highest beam energy (35 keV) is used as the original profile. The amorphous damage profile at a lower energy can be obtained using the linear relationship between the damage depth and the beam energy. Hence, the coefficients in Δ can be calculated by linear regression, and the results are shown in Table 1.

### 3.2. Determination of DPF Function Coefficients

**Figure 5:**
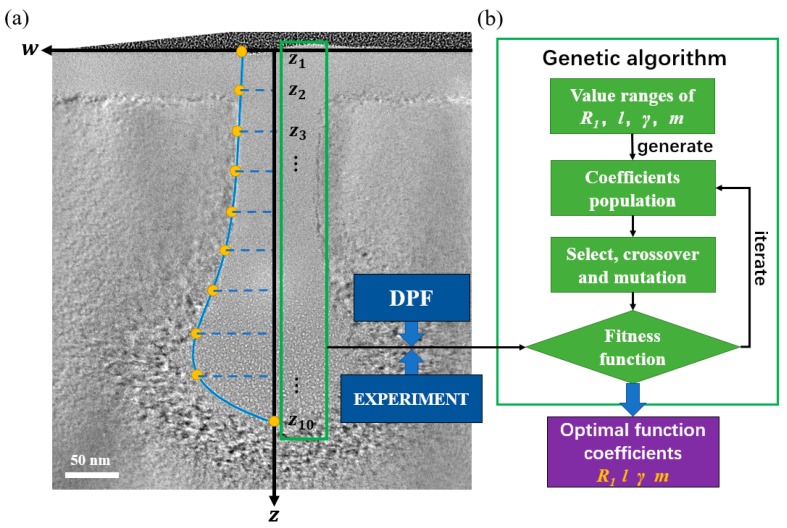
Process of genetic algorithm to solve DPF coefficients: (**a**) Coordinate system of the amorphous damage profile and the profile feature points; (**b**) Diagram of genetic algorithm to solve DPF coefficients.

After the establishment of the DPF, as shown in Figure 4, the calculation of remaining coefficients of the DPF was performed by a genetic algorithm. A set of experiments of He-FIB line scanning on a silicon substrate were performed at a beam energy of 35 keV with the ion dose sequentially increased from 0.01 nC/µm to 0.05 nC/µm at an interval of 0.01 nC/µm. For the damage profile of each ion dose, the genetic algorithm was used to determine the *R*_1_*^k^*, *l^k^*, *γ^k^* and *m^k^* coefficients of the DPF, where *k* is the index of each ion dose. As shown in Figure 5a, the coordinate system was established on each cross-section TEM image, and the amorphous damage profile at each ion dose was drawn. The amorphous damage depth was evenly distributed along the *z*-axis to obtain 10 profile feature points (*z_i_*, *w_i_*).

The profile feature points were implanted into the fitness evaluation as a reference standard. Hence, the fitness function based on the calculation error of DPF at half-width is as follows:

(10) $$fitness=c-\sum_{i=1}^{10}\left|w_{expt.}\left(z_{i}\right)-w_{DPF}\left(z_{i}\right)\right|,$$

where *c* is a constant. The group of *R*_1_*^k^*, *l^k^*, *γ^k^* and *m^k^* coefficients with high fitness are more likely to survive in population iterations. Figure 5b shows that each group of coefficient solutions of the DPF is defined as an individual in the program. A population with 50 groups of random coefficients is firstly generated within the range of these coefficients. The population is iterated in binary code through genetic operations of selection, crossover, and mutation. The program gives the best coefficients when the maximum number of iterations is met.

**Figure 6:**
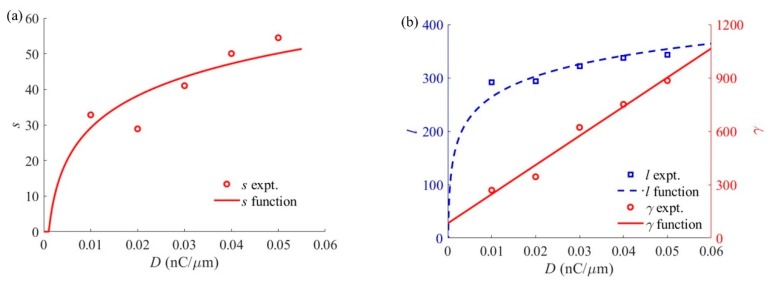
Variation of the DPF coefficients with ion dose at a beam energy of 35 keV: (**a**) Coefficient *s*; (**b**) Coefficients *l* and *γ*.

After solving five groups of *R*_1_*^k^*, *l^k^*, *γ^k^* and *m^k^* coefficients, Equations (4) and (6)–(8) are used to fit the relationship between the coefficients and process parameters, and the fitting results are shown in Table 1. Figure 6 shows the variations of the coefficients *s*, *l*, and *γ* with respect to the ion dose.

## 4. Results and Discussion

**Figure 7:**
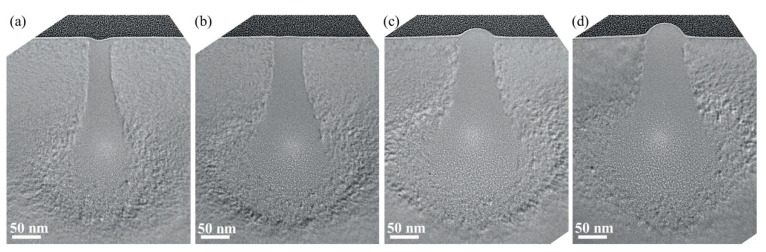
TEM images of cross-section samples of silicon substrates treated with He-FIB at a beam energy of 25 keV: (**a**) Ion dose of 0.02 nC/μm; (**b**) Ion dose of 0.03 nC/μm; (**c**) Ion dose of 0.04 nC/μm; (**d**) Ion dose of 0.05 nC/μm.

Figure 7 shows TEM images of cross-section samples of single-crystalline silicon treated with He-FIB with ion doses of 0.02, 0.03, 0.04 and 0.05 nC/μm at a beam energy of 25 keV. A 10 μm aperture was selected and used during the experiments. The beam spot of the experiments provided by equipment was 0.5 nm. However, the beam distribution is affected by several aspects, including the state of GFIS tip, the state of aperture, and adjustment of the processing parameter. Due to the effects of the aspects above, the surface damage width of the 25 keV group is shorter than that of other groups. As we can see from the TEM images, the amorphous damage profile gradually expands outward with an increasing ion dose.

**Figure 8:**
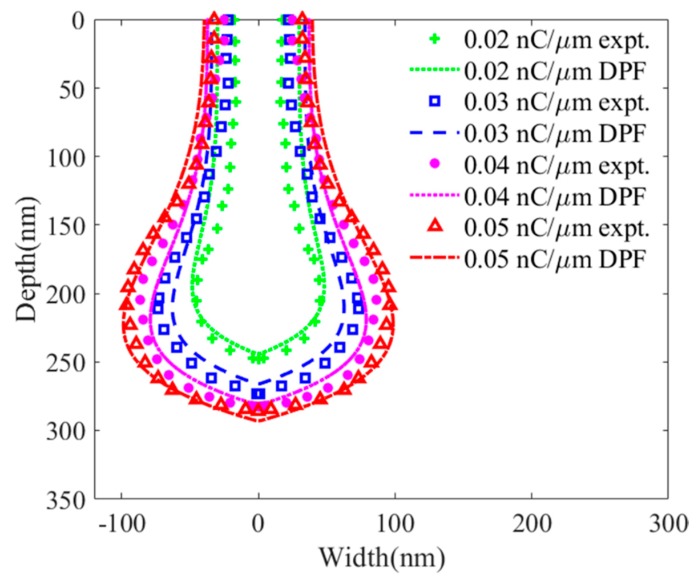
Experiments and DPF calculations of the amorphous damage profile of silicon substrate treated with He-FIB at a beam energy of 25 keV with ion doses of 0.02, 0.03, 0.04 and 0.05 nC/μm.

Figure 8 reports the DPF calculations and experimental results of damage induced by He-FIB with ion doses of 0.02, 0.03, 0.04 and 0.05 nC/μm at a beam energy of 25 keV. There are large errors in the high-energy damage region calculated by DPF, whose predicted profile is larger than the experimental profile. The calculated error of the high-energy damage region decreases with increasing ion dose. The DPF calculations of the low-energy damage region are smaller than the experimental results. Similarly, the calculated error of the low-energy damage region decreases with increasing ion dose. The calculation result of DPF is most accurate when the ion dose is 0.05 nC/μm. As the ion dose increases, the results of the experiments and DPF calculations tend to be consistent.

**Figure 9:**
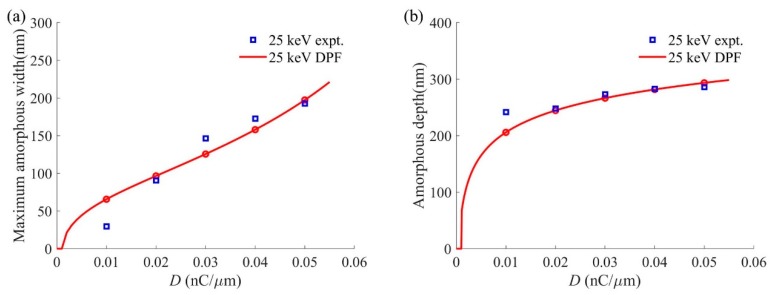
Comparison of experiments and DPF calculations of the amorphous damage profile characteristic dimensions of silicon substrate treated with He-FIB at a beam energy of 25 keV: (**a**) Maximum amorphous width; (**b**) Amorphous depth.

Figure 9a compares the maximum amorphous widths of the amorphous damage profiles calculated by DPF and measured through experiments, and Figure 9b does the same for the amorphous depth. Both the maximum amorphous width and amorphous depth increase with the increase in ion dose. As Figure 9a shows, the calculated error of the maximum amorphous width can fluctuate up to 35 nm with the ion dose. The calculated error of maximum amorphous width decreases as the ion dose increases and reaches a minimum of 5 nm when the ion dose is 0.05 nC/μm. As can be seen in Figure 9b, the simulation of the amorphous depth is very close to the experimental data when the ion dose is greater than 0.01 nC/μm.

**Figure 10:**
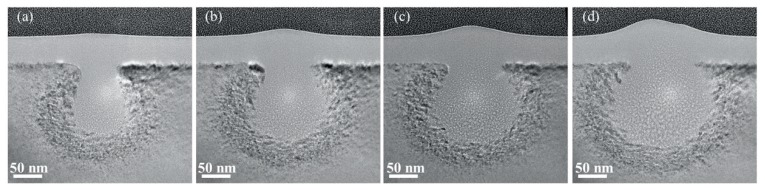
TEM images of cross-section samples of silicon substrate treated with He-FIB at a beam energy of 15 keV: (**a**) Ion dose of 0.02 nC/μm; (**b**) Ion dose of 0.03 nC/μm; (**c**) Ion dose of 0.04 nC/μm; (**d**) Ion dose of 0.05 nC/μm.

Figure 10 shows TEM images of cross-section samples of single-crystalline silicon treated with He-FIB with ion doses of 0.02, 0.03, 0.04 and 0.05 nC/μm at a beam energy of 15 keV. As mentioned earlier, a reduction in beam energy will result in a decrease in the damage depth. At the same ion dose, the reduction in the beam energy will slightly reduce the maximum amorphous width. Within the given range of beam energy variation, a slight change in maximum amorphous width has no significant influence on the amorphous damage prediction. Hence, the effect of beam energy on the maximum amorphous width is not introduced in the DPF.

**Figure 11:**
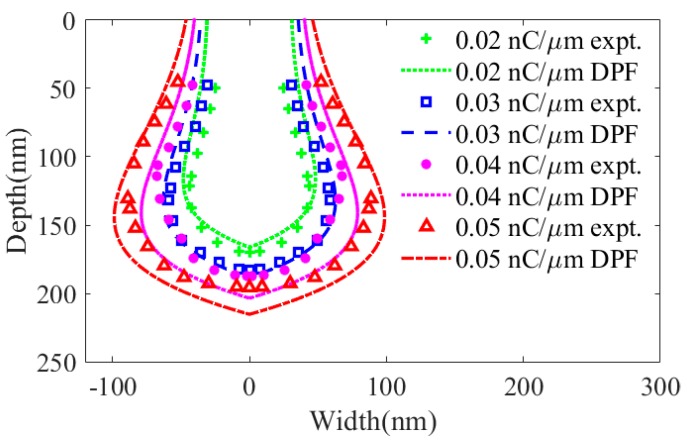
Experiments and DPF calculations of the amorphous damage profile of silicon substrate treated with He-FIB at a beam energy of 15 keV with ion doses of 0.02, 0.03, 0.04 and 0.05 nC/μm.

Figure 11 reports the DPF calculations and experimental results of damage induced by He-FIB with ion doses of 0.02, 0.03, 0.04 and 0.05 nC/μm at a beam energy of 15 keV. As can be seen from the figure, the DPF calculations have a clear trend, which proves that this function can simulate the changes of amorphous damage. Due to the influence of the equipment, compared with the amorphous damage profile induced by He-FIB with an ion dose of 0.03 nC/μm, the amorphous damage profile induced by He-FIB with an ion dose of 0.04 nC/μm does not change significantly. When the ion dose is 0.02 nC/μm, the low-energy damage region range calculated by DPF is smaller than the experimental result, and the high-energy damage region calculated by DPF is larger than the experimental result. Both results calculated by DPF are larger than the experimental results when the ion dose reaches 0.04 and 0.05 nC/μm. Aside from the sampling error of the profile, the fitting of the relationship between the DPF coefficients and the process parameters seriously affects the calculation accuracy. It is possible to carry out calibration experiments with a wide range of process parameters in order to enhance the fitting quality to improve the prediction accuracy.

**Figure 12:**
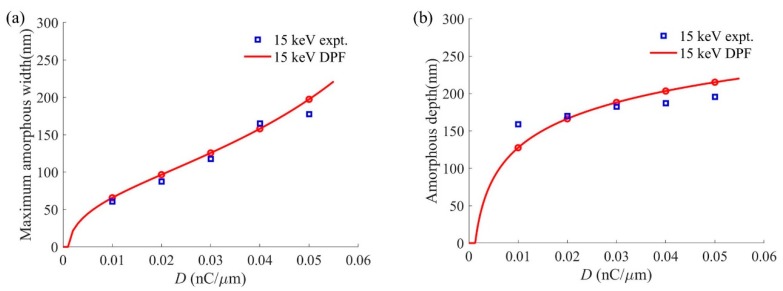
Comparison of experiments and DPF calculations of the amorphous damage profile characteristic dimensions of silicon substrate treated with He-FIB at a beam energy of 15 keV: (**a**) Maximum amorphous width; (**b**) Amorphous depth.

Figure 12 compares the maximum amorphous width and amorphous depth of the amorphous damage profile calculated by DPF and experiments. As Figure 12a shows, the maximum amorphous width has an approximately linear increase, and its maximum calculated error does not exceed 20 nm. There is a good agreement between the calculations of the maximum amorphous width and the experiments. From Figure 12b, we can see that the amorphous depth calculated by DPF increases sharply as the ion dose ranges from 0 to 0.01 nC/μm. Then, the growth trend gradually decreases when the ion dose exceeds 0.01 nC/μm. The calculated error of the amorphous depth decreases from 30 nm to the minimum of 4 nm when the ion dose reaches 0.02 nC/μm, and then gradually increases to 20 nm. The experiments show that the amorphous depth increases linearly, but the amorphous depth cannot be increased indefinitely. The results of the DPF calculation tend to be stable when the ion dose is large.

**Figure 13:**
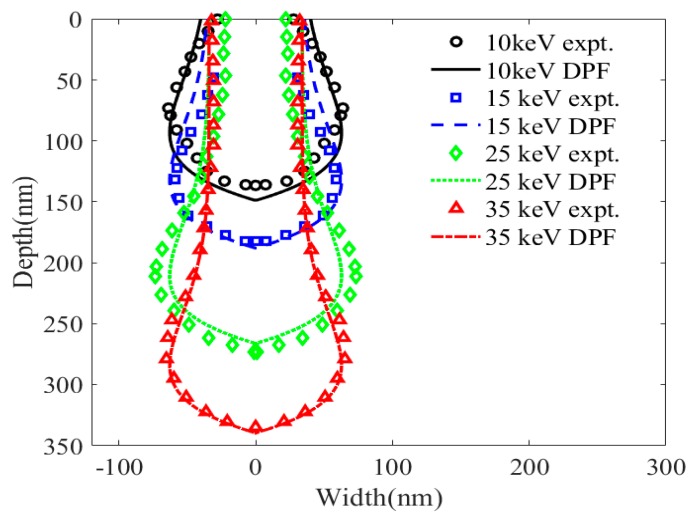
Experiments and DPF calculations of the amorphous damage profile of silicon substrates treated with He-FIB with an ion dose of 0.03 nC/μm at beam energies of 10, 15, 25 and 35 keV.

Figure 13 shows the DPF calculations and experiments of He-FIB incident on single-crystalline silicon at beam energies of 10, 15, 25 and 35 keV with an ion dose of 0.03 nC/μm. The DPF can accurately simulate the amorphous damage profile of single-crystalline silicon treated with He-FIB under different beam energies. Compared with the experiment profiles at 15 and 35 keV, when the ions with different initial energies decrease to the same energy value, the degree of lateral diffusion of ions increases with increasing energy. This will cause a large error when the DPF calculates the profile of the high-energy damage region. Because the DPF does not introduce the influence of lateral diffusion, the calculated width of the high-energy damage region profile at 15 keV is larger than the experimental width at 35 keV.

In Figure 13, the calculated error of the damage depth is highest at a beam energy of 10 keV. This is because the damage depth is also affected by lateral diffusion. The damage depth scaling coefficient Δ in DPF does not consider the effect of lateral diffusion. Therefore, the depth error increases as the beam energy decreases. The effect of the lateral diffusion can be introduced to improve the accuracy of the DPF in the future.

## 5. Conclusions

This study aimed to solve the problem that it is difficult to determine the range of amorphous damage in silicon substrates treated with line scanning by He-FIB. Based on the calibration experiments and the interaction between ions and solids, we proposed an experiment-based amorphous damage profile function that can predict the amorphous damage profile by inputting the beam energy, ion dose, and beam spot.

The genetic algorithm was utilized to solve the DPF coefficients corresponding to the amorphous damage profile with ion doses of 0.01–0.05 nC/μm at a beam energy of 35 keV. According to the variation of the coefficient, the form of the coefficient function was designed, and the relationships between the DPF coefficients and the process parameters were established. We conducted experiments of He-FIB incident on single-crystalline silicon with beam energies of 10, 15, 25 and 35 keV with ion doses of 0.01–0.05 nC/μm. When the ion dose was less than 0.01 nC/μm, the amorphous damage appeared as a groove with a large aspect ratio. Amorphous damage showed obvious segmental characteristic, and its damage range increased with ion dose when the ion dose exceeded 0.01 nC/μm. The experiments also showed that the larger depth of the high-energy damage region of the amorphous damage was induced by higher energy He-FIB.

The calculated results showed that the DPF can accurately simulate the amorphous damage profile of the single-crystalline silicon substrate treated with He-FIB at different ion beam energies with ion doses above 0.01 nC/μm. The DPF can describe the changes in the amorphous damage region caused by changes in beam energy and ion dose. In the future, more optimization measures can be introduced to improve the DPF’s calculation accuracy. This function has application potential and can be extended to light ion beam processing damage predictions of semiconductor substrate materials, such as gallium nitride and aluminum nitride.

## Figures and Tables

**Table 1:** Coefficients of DPF function for the single-crystalline silicon.

Coefficient	Value	Coefficient	Value
*α*_1_	1.792	*α*_2_	−0.112
*α*_3_	13.01	*α*_4_	89.08
*β*_1_	55.83	*β*_2_	521.2
*β*_3_	16380	*β*_4_	82.68
*β*_5_	−88.2	*β*_6_	9.504
*β*_7_	−7.831	*β*_8_	269.2
